# Supplementary material for: Adaptation of Methylobacterium extorquens to alternating carbon sources identifies the regulator CstR as an intersectional hub of cellular carbon metabolic dynamics and stress response
Source: bioRxiv. 2026 Jul 1:2026.06.30.735679. Preprint. [Version 1] doi: 10.64898/2026.06.30.735679 (PMC13344952; doi:10.64898/2026.06.30.735679)
Supplement: Supplement 1 [file NIHPP2026.06.30.735679v1-supplement-1.pdf]

bioRxiv preprint doi: https://doi.org/10.64898/2026.06.30.735679; this version posted July 1, 2026. The copyright holder for this preprint (which was not certified by peer review) is the author/funder, who has granted bioRxiv a license to display the preprint in perpetuity. It is made available under a CC-BY 4.0 International license.

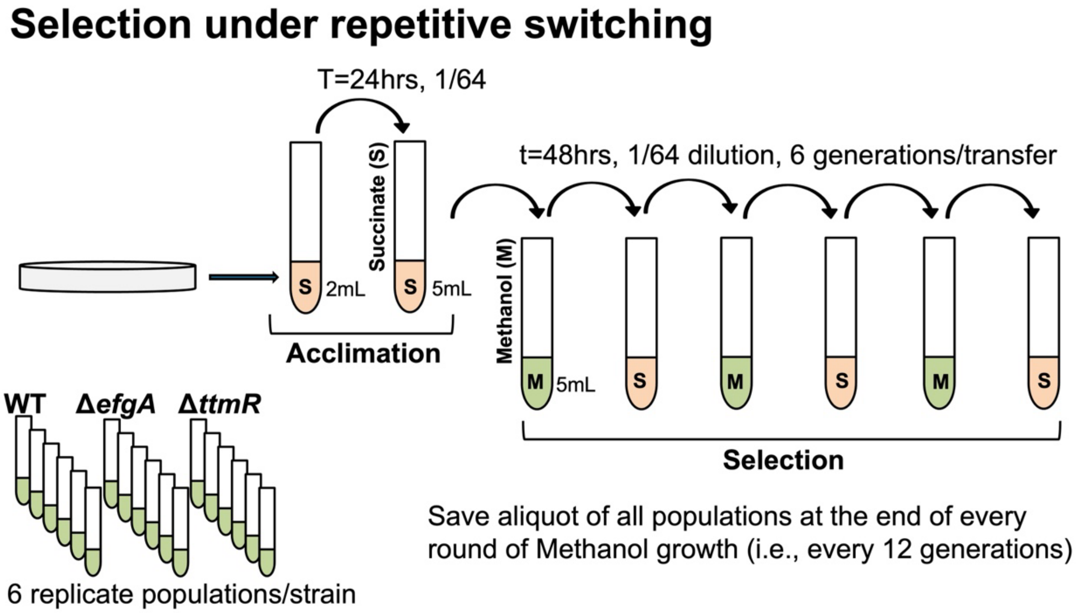

Figure S1. Selection regime of *M. extorquens* switching to and from methylotrophic growth. *M. extorquens* was experimentally evolved in MP liquid media for 174 generations (29 transfers). For each of three strains used (WT, Δ*efgA*, Δ*ttmR*), six independent replicate populations were used. Initial growth conditions relied on 3.5 mM succinate as a sole source of carbon and energy to acclimate cells to this mode of growth. At the first transfer, stationary phase cells were subcultured (1/64) into fresh media containing 15 mM methanol as a sole source of carbon and energy, transitioning their metabolic mode to methylotrophy. Populations were then continuously transferred between succinate and methanol-based growth at 48-72 hr intervals (approximately 6 generations). The resulting populations were sampled and saved periodically to assess genetic and phenotypic traits. Abbreviations: M, methanol; S, succinate.

bioRxiv preprint doi: https://doi.org/10.64898/2026.06.30.735679; this version posted July 1, 2026. The copyright holder for this preprint (which was not certified by peer review) is the author/funder, who has granted bioRxiv a license to display the preprint in perpetuity. It is made available under a CC-BY 4.0 International license.

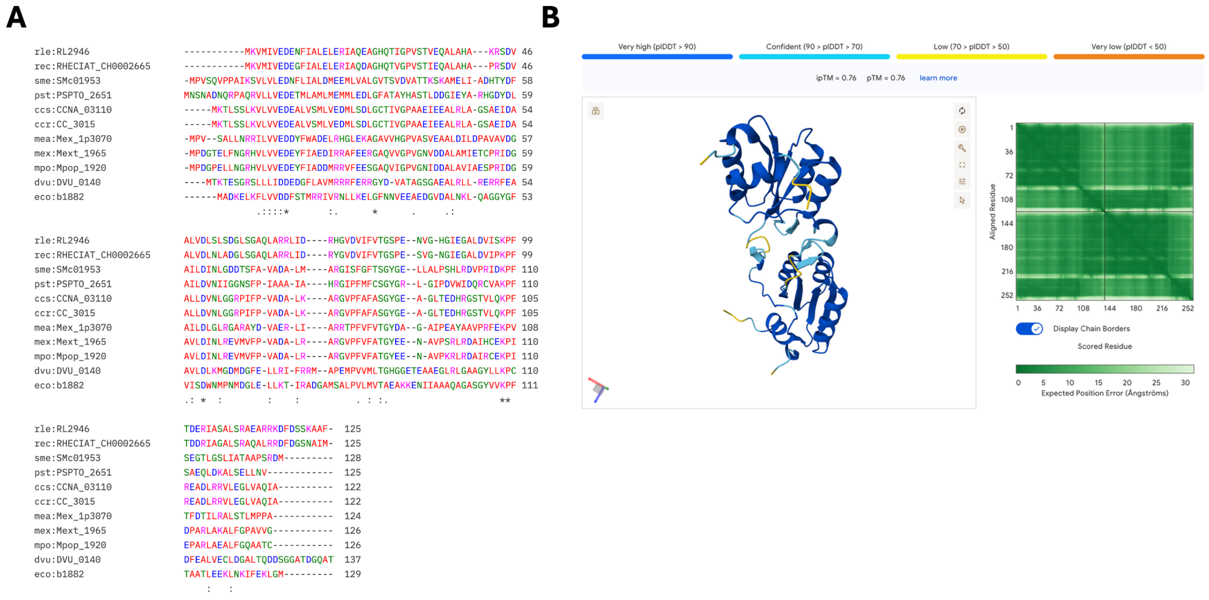

Figure S2. Alignments of the CstR protein with homologs A) A Clustal Omega (26) generated multiple sequence alignment of CstR from *M. extorquens* PA1 (mex) and high similarity homologs from *M. extorquens* AM1 (mea), *M. populi* (mpo), *Caulobacter vibrioides* NA1000 (ccs), *Caulobacter vibrioides* CB15 (ccr) MrrA protein, *E. coli* K-12 MG1655 (eco) CheY protein, *Rhizobium johnstonii* 3841 (rle), *Rhizobium etli* CIAT 652 (rec), *Sinorhizobium meliloti* 1021 (sme), *Pseudomonas syringae* pv. tomato DC3000 (pst), *Nitratidesulfovibrio* (formerly *Desulfovibrio*) *vulgaris* Hildenborough (dvu) Rrf1 protein, and a high similarity Rrf1 homolog from *Methylobacterium* sp001542815. Conservation of residues is indicated when identical (*), strongly similar (:), or weakly similar (.). Small-hydrophobic residues (less Y) are in red (AVFPMILW), acidic residues are in blue (DE), basic residues are in magenta (RHK), and hydroxyl + sulfhydryl + amine + G residues are in green (STYHCNGQ). B) A structural model of CstR predicted with AlphaFold3. Displayed is a picture of the best scored model generated.

bioRxiv preprint doi: https://doi.org/10.64898/2026.06.30.735679; this version posted July 1, 2026. The copyright holder for this preprint (which was not certified by peer review) is the author/funder, who has granted bioRxiv a license to display the preprint in perpetuity. It is made available under a CC-BY 4.0 International license.

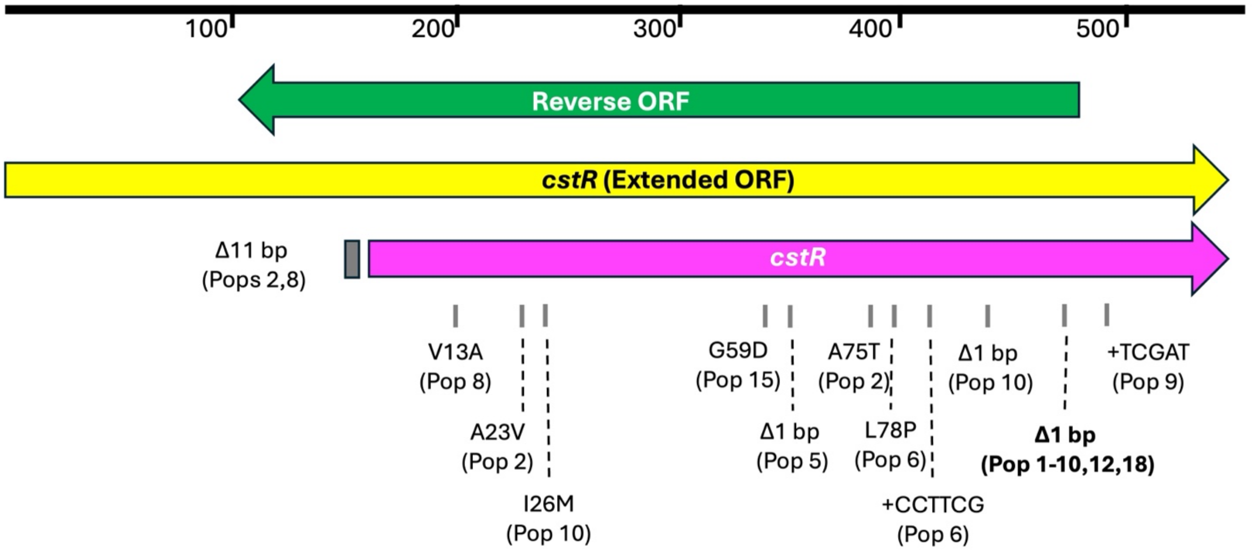

Figure S3. Genomic region near *cstR*. The genomic region containing *cstR* and nearby sequence is shown, with the pink arrow depicting the annotated gene, the yellow arrow depicting the extended *cstR* ORF that contains an additional 162 bp upstream of the annotated *cstR* start codon, and the green arrow depicting an ORF oriented antiparallel but overlapping a large portion of *cstR*. Gray boxes and dashes indicate the location of identified *cstR* mutations among the experimentally evolved populations (see **Table 1**). The mutation referred to throughout the text as *cstR*^{evo} is indicated in bold.

bioRxiv preprint doi: https://doi.org/10.64898/2026.06.30.735679; this version posted July 1, 2026. The copyright holder for this preprint (which was not certified by peer review) is the author/funder, who has granted bioRxiv a license to display the preprint in perpetuity. It is made available under a CC-BY 4.0 International license.

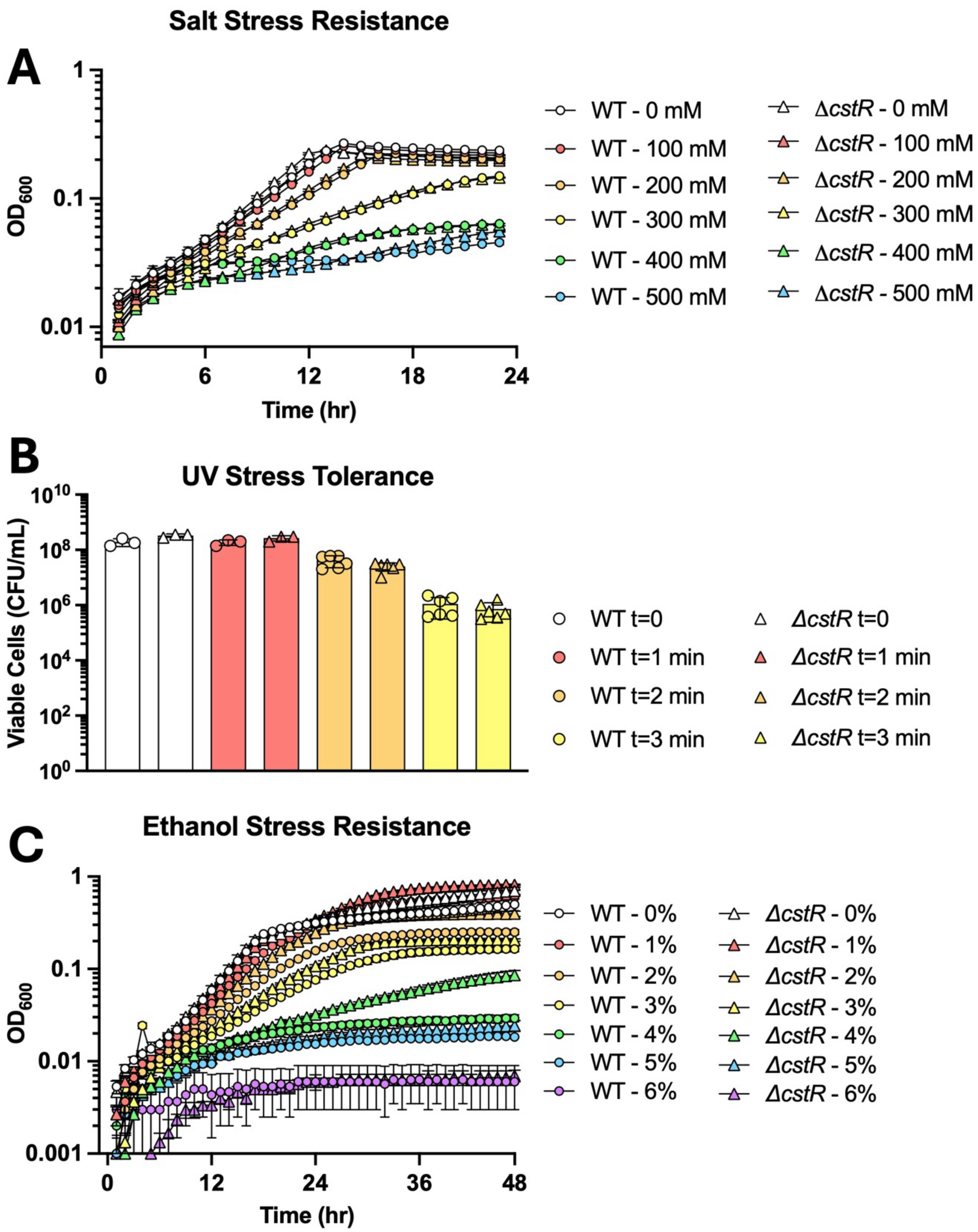

Figure S4. CstR does not impact resistance/tolerance to all stressors. WT and Δ*cstR* strains were exposed to A) a range of salt (sodium chloride) concentrations (growth), B) different time exposure to ultraviolet radiation (survival), and C) a range of ethanol concentrations (growth). Strains were treated in biological triplicate; points represent the mean and error bars represent standard error of the mean (SEM).

bioRxiv preprint doi: https://doi.org/10.64898/2026.06.30.735679; this version posted July 1, 2026. The copyright holder for this preprint (which was not certified by peer review) is the author/funder, who has granted bioRxiv a license to display the preprint in perpetuity. It is made available under a CC-BY 4.0 International license.

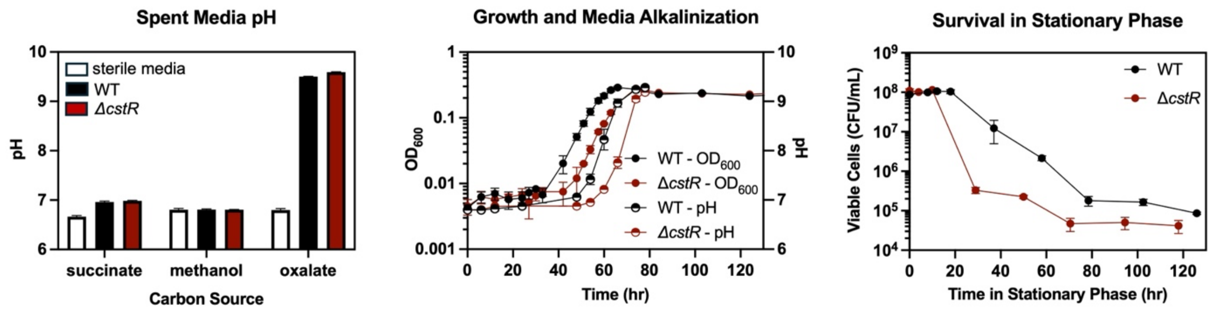

Figure S5. Growth and survival in oxalate media. A) Stationary phase media pH values were measured from MP media following cell growth of WT and Δ*cstR* strains upon either succinate, methanol, or oxalate as the sole carbon source. B) Percentage of population surviving in stationary phase of WT and Δ*cstR* strains in the same media as panel A, measured at 72 hr of growth. C) Oxalate-grown cells (that lost viability in stationary phase) were subcultured in oxalate media and demonstrate an offset in growth (filled circles) due to different starting inoculum resulting from differential die-off; supernatant pH (half-filled circles) was monitored over time to demonstrate alkalinization of media during active growth. D) Viability in spent, alkalinized media was measured once cultures reached stationary phase (determined by observing a measured peak/non-change in OD_{600}). Plotted values represent a mean of four biological replicates, and error is represented by SEM.

bioRxiv preprint doi: https://doi.org/10.64898/2026.06.30.735679; this version posted July 1, 2026. The copyright holder for this preprint (which was not certified by peer review) is the author/funder, who has granted bioRxiv a license to display the preprint in perpetuity. It is made available under a CC-BY 4.0 International license.

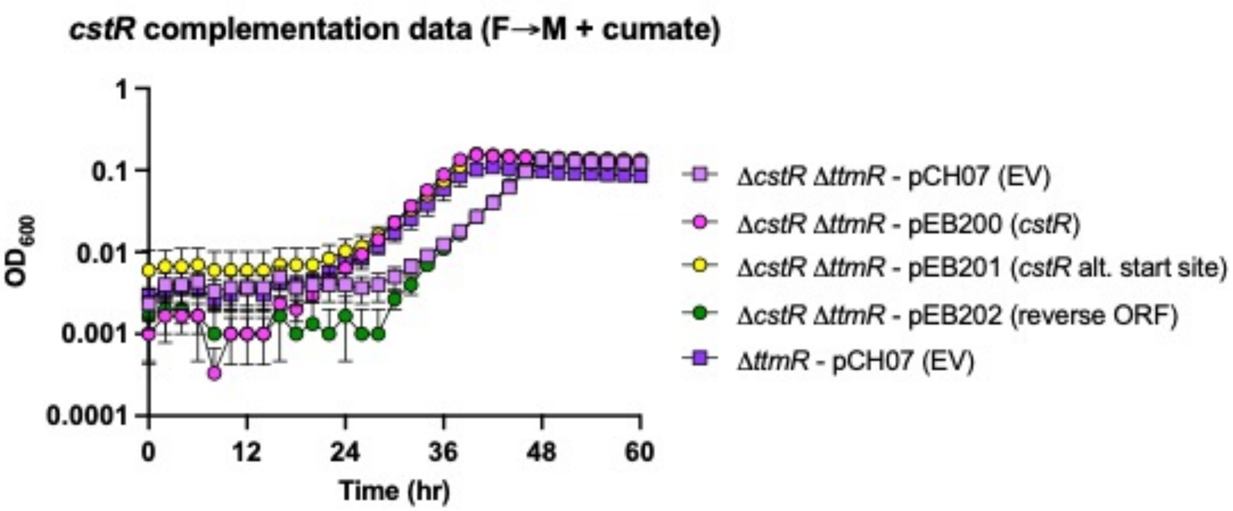

Figure S6. Complementation of CstR function is provided by the annotated CDS. Growth of strains lacking *ttmR* or *ttmR* and *cstR* and harboring a kanamycin-resistant expression plasmid under inducing conditions (containing cumate) was tracked during a formate-to-methanol transition. In addition to empty vector (EV) controls, plasmids containing a) annotated *cstR* (381 bp, pEB200), the extended *cstR* ORF (543 bp, pEB201), and a reverse ORF overlapping *cstR* (375 bp, pEB202); these ORFs are also visualized in **Figure S3**. Points represent the means of biological triplicates and error bars represent SEM.

bioRxiv preprint doi: https://doi.org/10.64898/2026.06.30.735679; this version posted July 1, 2026. The copyright holder for this preprint (which was not certified by peer review) is the author/funder, who has granted bioRxiv a license to display the preprint in perpetuity. It is made available under a CC-BY 4.0 International license.

Table S1. Bacterial strains and plasmids.

Table S2. Population whole genome sequencing results.

Table S3. Differentially expressed genes in the Δ*cstR* background vs. WT *M. extorquens*. The following list of genes were differentially regulated, as determined by a statistically significant change (FDR < 0.01) between *ΔcstR* and WT *M. extorquens* PA1 strains during growth with succinate as a sole carbon source or during the transition to methylotrophy (methanol as sole carbon source).

Table S4. Gene enrichment among differentially expressed genes in the Δ*cstR* strain.
